# Cavitation enhancement by organic additives: a systematic study probing the universal effect of additive vapor-pressure

**DOI:** 10.1016/j.ultsonch.2026.108043

**Published:** 2026-09-08

**Authors:** Chunhua Wei, Yuying Yang, Guangyu He, Zhouling Wu, Hongyu Chen, Xueyang Liu

**Affiliations:** a Institute of Advanced Synthesis (IAS) and School of Chemistry and Molecular Engineering, Jiangsu National Synergetic Innovation Centre for Advanced Materials, Nanjing Tech University, Nanjing 211816, China; b Department of Chemistry, School of Science, Research Center for Industries of the Future, Westlake University, Hangzhou 310030, China; c Institute of Natural Sciences, Westlake Institute for Advanced Study, Hangzhou 310024, China

**Keywords:** Ultrasonic cavitation, Organic solvents, Vapor pressure, Chromogenic reagents, Reactive oxygen species

## Abstract

Carbon tetrachloride (CCl_4_) is a common additive for enhancing ultrasonic cavitation, and the governing mechanism is generally attributed to its chemical reactivity. Here, we systematically investigate chlorinated solvents, insoluble alkanes, and soluble alcohols as additives in ultrasonication, using complementary co-sonication and stepwise sonication protocols, alongside [Fe(SCN)_6_]^4−^ and DPD as chromogenic probes. Unexpectedly, all additive categories exhibit a common volcano-shaped dependence of cavitation intensity on additive vapor pressure, irrespective of chlorine content or water solubility, indicating that vapor pressure rather than intrinsic chemical reactivity dominates cavitation enhancement. Stepwise sonication decouples cavitation from chromogenic reactions and reveals that chlorinated additives predominantly generate free chlorine whereas chlorine-free additives mainly produce peroxides. Based on these observations, we propose that vapor pressure facilitates bubble nucleation and at excessive values, weakens bubble collapse, leading to a peak with optimum cavitation efficiency. This work establishes a unified physical framework for understanding cavitation promotion by organic additives and provides guidance for the rational design of efficient sonochemical systems.

## Highlights

• Additive vapor pressure, in addition to its chemical reactivity, serves as a critical factor regulating ultrasonic cavitation efficiency.
• Two chromogenic probes are applied in co- and stepwise sonication experiments to detect and assign reactive intermediates
• In ultrasonication, chlorinated additives produce free chlorine, and alkane/alcohol systems are dominated by peroxide-based reactive species
• [Fe(SCN)_6_]^4−^ and DPD suit different sonication modes with complementary advantages.

## 1 Introduction

Cavitation refers to the formation of vapor bubbles in a solution under low pressure. In daily life, erosion of marine propellers and ultrasonic cleaning are the typical phenomena that are closely related to the effects of cavitation. To control cavitation, it's essential to understand its mechanisms.

Ultrasonication is the primary research method for studying cavitation in laboratories, because other methods, such as propeller erosion, are cumbersome and less controllable in a laboratory setting. As ultrasound passes through a solution, it creates periodic tension and compression. When the ultrasonic intensity exceeds the solution's tensile strength, local separation occurs to form cavitation bubbles [1]. These cavitation bubbles grow under alternating positive and negative pressure, and upon reaching a critical size, will collapse and implode, creating transient high temperature, high pressure, shock waves, etc [1], [2], [3], [4], [5].

There are mechanical and chemical effects as consequences of imploding cavitation bubbles. The former results from the generation of shock waves upon bubble collapse and the formation of high-speed microjets as the collapse occurs asymmetrically, and their impacts can be examined by the resulting mechanical damage, such as erosion of aluminum foil [6], [7], [8], but it is generally difficult to separate their respective contributions. We have previously monitored the extent of bending and breaking of Ag nanowires [9] to assess the cavitation effects under various circumstances. We could even infer the effective radius of shock waves from the unidirectional multiple bends (coplanar *cis*-conformation) of Ag nanowires.

On the other hand, the primary source of the chemical effects is the reactive chemical species, such as hydroxyl radicals, generated from the pyrolysis of water molecules under the high-temperature and high-pressure conditions of cavitation implosion. Several approaches are available for quantifying the derivatives of hydroxyl radicals [10], [11], [12], serving as an indirect means to evaluate acoustic cavitation effects, including fluorometric and colorimetric methods. The former uses luminol to react with radicals, whereby the cavitation effect is assessed by measuring the resulting chemiluminescence intensity [7], [13], [14]. Since this luminescence intensity is proportional to the number of reacting radicals at a given moment, it mainly reflects the transient concentration of free radicals. This method enables visualization of the spatial distribution of cavitation effects [7], [15], [16], but the dynamic attenuation of chemiluminescence intensity is particularly difficult to quantify during continuous ultrasonication. In comparison, the colorimetric method uses the free radicals to oxidize Fe^2+^ to Fe^3+^, which form a red complex with thiocyanate ions in solution [17], [18]. The values measured by the colorimetric method represent the cumulative oxidation effect of free radicals over the entire ultrasonication process and thus, they reflect the overall chemical effects originating from cavitation implosions.

In many of the literature studies on cavitation effects, including fluorometric and colorimetric methods, CCl_4_ is added as a promoting additive. Despite its widespread use, no unified understanding has been reached regarding the role of CCl_4_. In one view, CCl_4_ may generate radicals more easily than water upon implosion [19], [20], [21], [22]; in another, CCl_4_ may react with the initial radicals [23], [24], [25], [26], [27] to form stabler Cl-based radicals, thus increasing the effective concentration of oxidants. In other words, the existing arguments mainly focused on the chemical reactivity of CCl_4_.

Our research group has long investigated the nucleation and growth of nanoparticles, either solid [28] or liquid [29] ones, where the probability of nucleation depends critically on the oversaturation of growth materials. From our perspective, the formation of cavitation bubbles from a homogeneous solution also represents a form of homogeneous nucleation, and therefore follows analogous fundamental principles. More specifically, cavitation constitutes a complex multi‑step phenomenon, governed by the probability of homogeneous nucleation (*P*_nucleation_), the probability of implosion (*P*_implosion_), the probability of radical formation upon implosion (*P*_radical_), and the subsequent competing chemical reactions (*P*_reaction_). These processes proceed as largely independent, sequential steps, and each contributes substantially to the final radical concentration. On a first-order approximation, the overall effect should be the product of individual stepwise probabilities (Eq. (1)).

(1) $$\begin{array}{c} E_{\mathrm{overall}}=P_{\mathrm{nucleation}}\times P_{\mathrm{implosion}}\times P_{\mathrm{radical}}\times P_{\mathrm{reaction}} \end{array}$$

More recently, we compared the efficiency of positive- and negative-curvature nanoparticles in terms of promoting cavitation by using a colorimetric method [18]. The results indicate that solid Au nanoparticles facilitate the initial bubble nucleation and thus enhance the overall effects. In other words, the first step *P*_nucleation_ likely plays a critical role. On this basis, we speculate that the chemical reactivity of CCl_4_ may not be the only factor.

Here, we systematically studied the influence of organic-solvent additives on ultrasonic cavitation performance using a colorimetric method. We found that cavitation enhancement is a general effect for organic additives, regardless of water solubility or the presence of chlorine atoms. Based on the characterization of long-lived intermediates, we demonstrate two distinct reaction mechanisms, mediated by free chlorine and peroxyl species, respectively. Importantly, in all systems the observed color change correlates more strongly with solvent vapor pressure than with intrinsic chemical reactivity. Hence, we postulate that the vapor pressure of organic additives plays a critical role in facilitating the initial cavitation nucleation step by lowering the corresponding activation barrier.

## 2 Materials and methods

### 2.1 Materials

All chemical reagents were used as purchased without further purification. Chlorobenzene (C_6_H_5_Cl, AR, 99.5%), 1,2-dichloroethane (C_2_H_4_Cl_2_, AR, 99.5%), carbon tetrachloride (CCl_4_, AR, 99.5%), dichloromethane (CH_2_Cl_2_, AR, 99.5%), hexane (CH_3_(CH_2_)_4_CH_3_, AR, 97.0%), ethylene glycol (HOCH_2_CH_2_OH, AR, 99.5%), 2-propanol ((CH_3_)_2_CHOH, AR, 99.7%), ethanol (CH_3_CH_2_OH, AR, anhydrous, 99.7%), methyl alcohol (CH_3_OH, GR, Anhydrous, 99.9%) were purchased from General-Reagent. O-chlorotoluene (C_7_H_7_Cl, AR), n-pentane (C_5_H_12_, 99.0%), 1-propanol (C_3_H_8_O, 99.5%) and sodium thiocyanate (NaSCN, 99.0%) were purchased from Macklin. Decane (C_10_H_22_, 98.0%) and cyclohexane (C_6_H_12_, 99.5%) were purchased from Energy Chemical. Trichloromethane (CHCl_3_, AR), hydrochloric acid (HCl, GR), sulfuric acid (H_2_SO_4,_ GR) and hydrogen peroxide 30% aqueous solution (H_2_O_2_, GR) were purchased from Sinopharm Chemical Reagent Co., Ltd. 1,1,2,2-Tetrachloroethane (C_2_H_2_Cl_4,_ 99.0%), Di-*tert*-butyl peroxide (C_8_H_18_O_2_, 98.0%) and N,N-Diethyl-*p*-phenylenediamine (C_10_H_16_N_2_, 99.0%) were purchased from Adamas-Beta. Sodium hypochlorite (NaClO, available chlorine 30.0%) was purchased from Meryer Shanghai Chemical Technology Co., Ltd. Ammonium iron(Ⅱ) sulfate (Fe(NH_4_)_2_(SO_4_)_2_, 99.0%) was purchased from Shanghai Haohong Biomedical Technology Co., Ltd. Silver nitrate (AgNO_3,_ 99%) was purchased from Sigma-Aldrich. Horseradish peroxidase (HRP, BR) was purchased from Shanghai Yuanye Bio-technology Co., Ltd.

### 2.2 Experimental setup

UV–Vis-NIR spectra were collected on a Mettler Toledo UV5. Ultrasound machine was the JY92-IIDN type Ultrasonic homogenizer (tip sonicator), with the maximum power of 900 W and 25 kHz (typical experiments were carried out at 180 W). The pH of all solutions was determined with a SevenDirect SD20 pH meter manufactured by Mettler Toledo.

### 2.3 Colorimetric method

In 25 mL of 10 mM Fe(NH_4_)_2_(SO_4_)_2_ solution, 2.0 mL of 1.2 M NaSCN solution was added, and the pH was adjusted to 2.0 with 0.70 mL HCl to produce a 9.0 mM solution of [Fe(SCN)_6_]^4−^. 0.10 g N,N-diethyl-p-phenylenediamine (DPD) was dispersed in 10 mL of 0.050 M H_2_SO_4_ solution to obtain DPD chromogenic reagent with a concentration of 60.0 mM.

## 3 Results and discussion

**Fig. 1:**
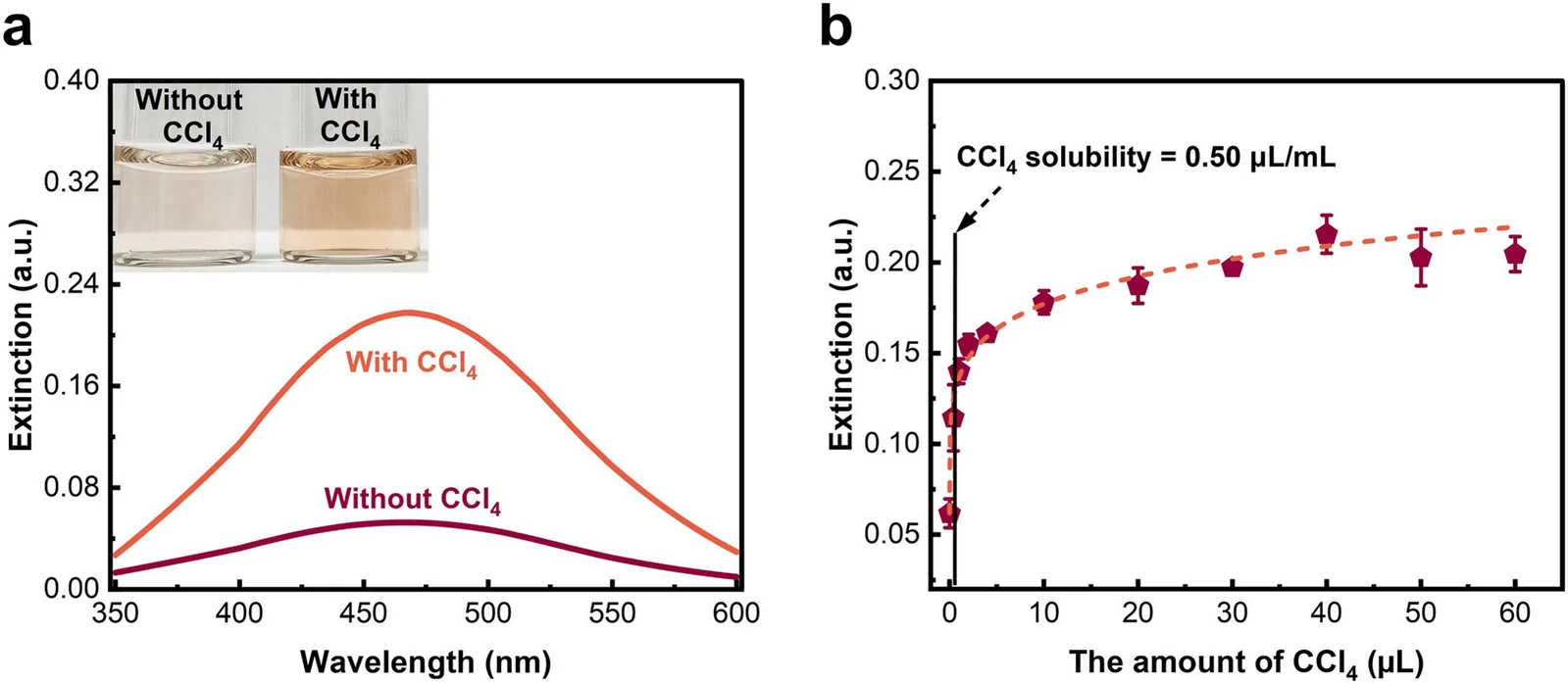
Case study on CCl_4_-based sonication experiments. (a) UV–Vis extinction spectra of the red complex [Fe(SCN)_6_]^3−^ formed after ultrasonication in the absence and presence of CCl_4_. The inset shows the corresponding visual color change of the solutions; (b) The extinction intensity of the red complex [Fe(SCN)_6_]^3−^ as a function of the amount of added CCl_4_ in a total volume of 2.0 mL (*i.e.*, 0–3.0% v/v). In the ultrasonic experiments, the total volume of the solution was fixed at 2.0 mL. The labeled value of 0.50 µL/mL corresponds to the solubility limit of CCl_4_ in water, meaning that a maximum of 1.0 µL of CCl_4_ can be dissolved in 2.0 mL of water under the experimental conditions. All samples were measured 3 times and error bars are included for all data points, with a few small ones blocked by symbols, same below. Note: The clear solution exhibits negligible scattering, so measured extinction approximates light absorption. Spectra are raw experimental data without normalization or concentration correction.

For ultrasonication tests, we prepared 9.0 mM [Fe(SCN)_6_]^4−^ solution by mixing solutions of Fe(NH_4_)_2_(SO_4_)_2_ and excess NaSCN. The pH of the solution was adjusted to 2.0 by adding HCl. The resulting mixture was placed in an ice bath and applied tip sonication (10 min at 25 kHz and 180 W, defined as the standard condition), so that the reaction temperature was kept constant (0 °C) despite the heat generated during the process. A slight reddish color was observed (Fig. 1a), indicating oxidation to form the red complex [Fe(SCN)_6_]^3−^. The extent of oxidation was readily quantified by measuring the characteristic extinction peak at 476 nm. This colorimetric method was adopted from the literature [17] and our previous work [18]. It is facile and the red color intensity correlates to the integrated sonication effects, as opposed to snapshots of transient effects.

As discussed in the introduction, CCl_4_ is often added as a promoter for sonication tests [13], [17], [18]. Indeed, upon addition of CCl_4_ (40 µL, corresponding to 2.0% v/v in a total volume of 2.0 mL) to the above sonication experiment, the resulting red color was obviously more intense (Fig. 1a). As CCl_4_ was increased from 0.40 to 10 µL, the resulting red color intensity (Fig. 1b, the 476 nm peak, same below) increased sharply and then plateaued from 10 to 60 µL. We note that this sharp increase coincides with the solubility limit of CCl_4_ (0.50 µL/mL). It is likely that the dissolved CCl_4_ is vaporized during cavitation, while excess CCl_4_ sustains the prolonged sonication process. The typical additive dosage of 40 µL (2.0% v/v) lies in the plateau region and is sufficient to compensate for the loss during sonication.

It is believed that CCl_4_ generates chlorine-based radicals during cavitation implosion, and these radicals are responsible for the chemical effects of sonication [13], [19], [20], [24], [30], [31]. Based on this mechanism, other organic solvents containing C-Cl bonds are expected to exhibit comparable promotion of the cavitation effect. As discussed in the introduction, two reaction pathways should be distinguished mechanistically. The radical-mediated route produces highly reactive radicals (*e.g.*, ⋅OH radicals), so that its subsequent reaction depends more on collision probability rather than the strength of C-Cl bonds. The other pathway is direct C-Cl bond cleavage, whose reactivity is directly governed by the C-Cl bond dissociation energy.

**Fig. 2:**
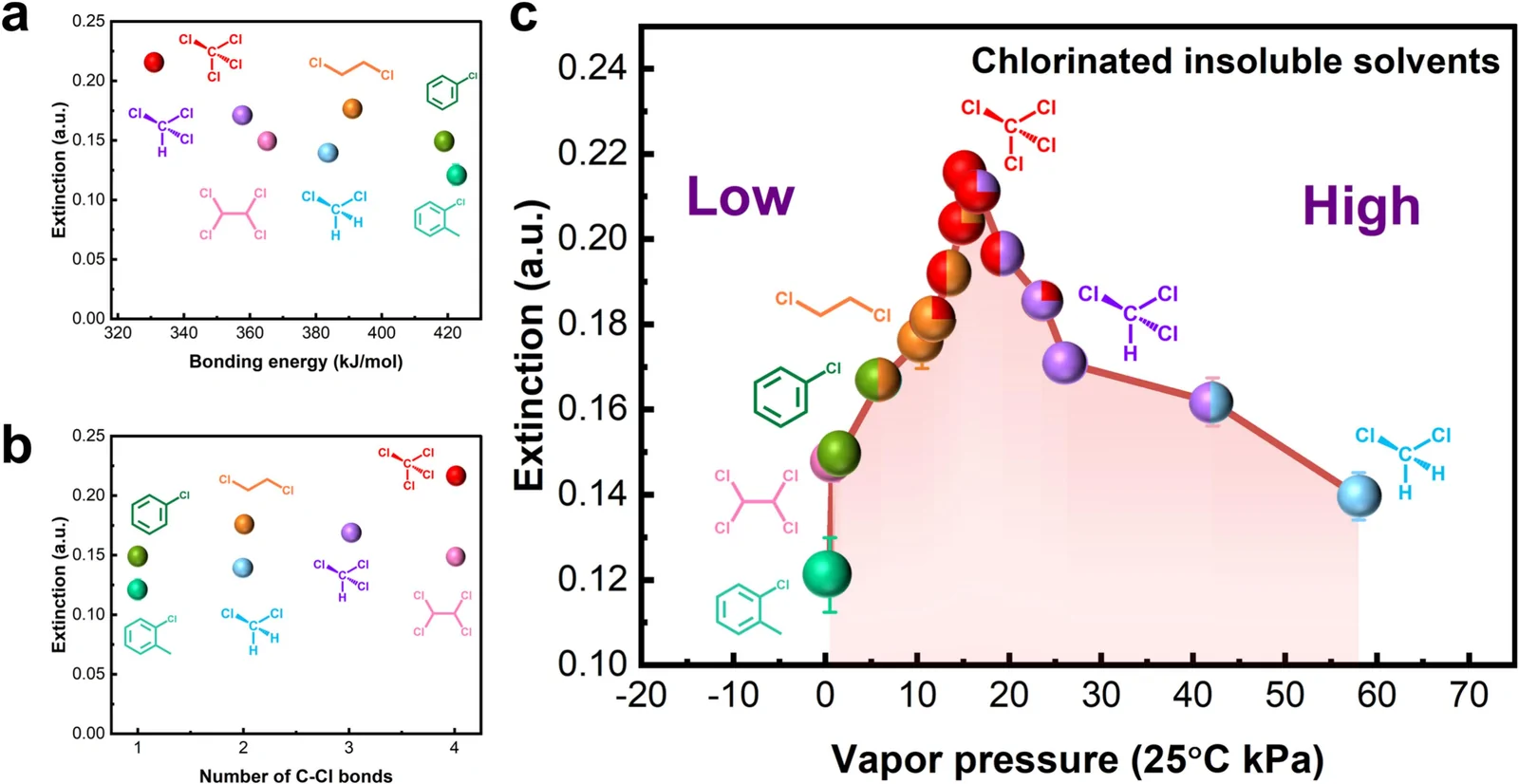
Correlation between cavitation performance (characterized by the red complex [Fe(SCN)_6_]^3−^) and the properties of organic additives. (a) Variation of extinction intensity with C-Cl bond dissociation energy (kJ/mol) for chlorinated additives. The bond energy calculation methods and results are included in the SI. (b) Extinction intensity as a function of the number of C-Cl bonds in chlorinated additives. Extinction intensity as a function of the vapor pressure (at 25 °C) of the respective solvent additives: (c) chlorinated insoluble solvents. The intermediate compositions were obtained by repeated binary (1:1, v/v) mixing of adjacent solvents. The same data with error bars are presented in SI.

To test these hypotheses, we sonicated aqueous solutions with Cl-containing organic additives under the standard conditions, including chlorobenzene, o-chlorotoluene, C_2_H_4_Cl_2_, CH_2_Cl_2_, CCl_4_, CHCl_3_, and C_2_H_2_Cl_4_. The amount of each organic additive was fixed at 40 µL (2.0% v/v). Considering probabilities, this promotional effect is anticipated to intensify with an increasing number of chlorine atoms within the molecular structure. However, as we plotted C-Cl bonds versus the resulting red color intensity, there was no clear correlation (Fig. 2b and S8), deviating from our expectation.

Furthermore, we correlated C-Cl bond dissociation energies with colorimetric signals using the same dataset. CCl_4_, which possesses the weakest C-Cl bonds among all tested additives, yielded the strongest signal, indicating that it is readily cleaved under cavitation conditions to release chlorine radicals. Although a weak negative correlation was observed, several samples deviated markedly from this trend (Fig. 2a and S8). This indicates that C-Cl bond energy alone cannot fully account for all experimental results; it is merely one contributing factor.

On the basis of the reasoning in the introduction, an alternative explanation is that the small amount of CCl_4_ may alter the system’s vapor pressure, lowering the barrier for forming vapor-filled bubbles. Despite its low fraction (40 µL, 2.0% v/v) and low solubility in water (0.05 wt%), the solution is well homogenized by sonication. Therefore, using the aforementioned dataset, we plotted the vapor pressure of these chlorinated solvents against the red color intensity of [Fe(SCN)_6_]^3−^ after sonication. The resulting Fig. 2c and S8 showed a clear trend: the cavitation effect initially increases and then decreases as the vapor pressure rises, giving a maximum at 15.3 kPa. Moreover, as we added data points by mixing a solvent with its neighbor at 1:1 v/v ratio, the resulting colorimetry-based cavitation effect was roughly at the middle, further supporting this trend. More points were added near the peak, and the two-way monotonical trends are unmistakable.

From our perspective, gas bubble nucleation is in principle analogous to the nucleation of solid [28] and liquid nanoparticles [29]. Specifically, nucleation constitutes the rate‑limiting step; after this step, subsequent growth only enlarges the pre‑existing nuclei. The nucleation probability, and thus the number of resulting nuclei and subsequent nanoparticles, depends on the energy barrier for forming an initial nucleus within a homogeneous solution.

Under ultrasonication, high-vapor-pressure additives make it easier to “push apart” the liquid phase and generate cavitation bubbles filled with additive vapor. This proliferation of cavitation bubbles leads to a marked increase in radical yield. As the vapor pressure of the additive decreases, such promotional effects gradually diminish. In this context, the “energy barrier” for liquid-phase distension and cavitation bubble nucleation is reduced, leading to an enhanced probability of cavitation (*P*_nucleation_). However, further elevation of vapor pressure reduces the specific heat ratio inside the bubble, which yields a lower implosion temperature and suppressed radical generation. This results in milder, less violent bubble collapse [3] and inhibits subsequent radical generation (*P*_radical_), ultimately weakening the overall cavitation performance.

These experiments confirm that vapor pressure, alongside the intrinsic chemical reactivity of CCl_4_, serves as a key determinant governing cavitation efficiency. Considering the critical role of additive vapor pressure, the same principle should apply broadly to organic additives.

**Fig. 3:**
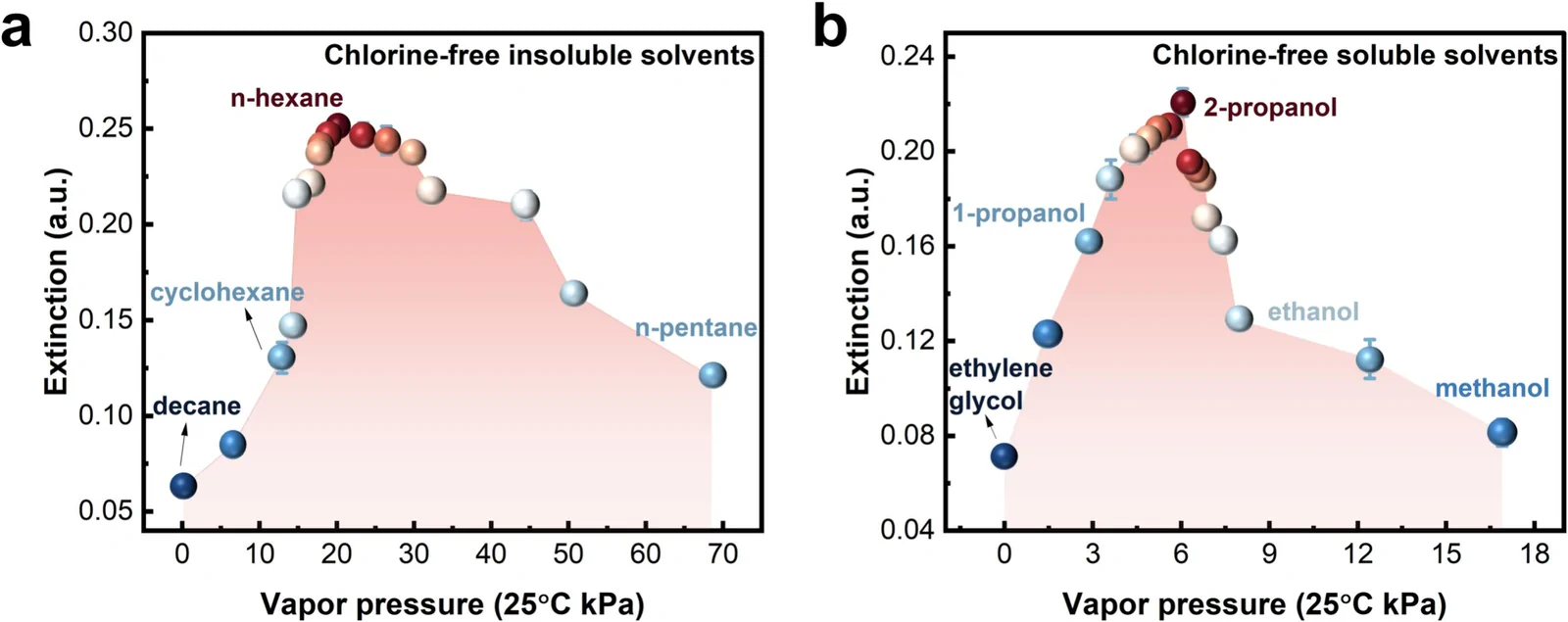
Correlation between cavitation performance (characterized by the red complex [Fe(SCN)_6_]^3−^) and the properties of organic additives. Extinction intensity as a function of the vapor pressure (at 25 °C) of the respective solvent additives: (a) alkane solvents, (b) alcohol solvents. Unlabeled data points represent intermediate solvent compositions obtained by successive 1:1 (v/v) binary mixing of adjacent pure solvents.

To verify this hypothesis, we first tested n‑hexane (40 µL, 2.0% v/v), a water‑insoluble solvent with high vapor pressure and no C-Cl bonds, and observed a pronounced red color enhancement (19.1%) relative to the control group, validating our hypothesis. Ultrasonic measurements with other water‑insoluble alkanes, including decane, cyclohexane, n‑hexane, and n-pentane, yielded similar promotional effects on cavitation. Fig. 3a and S8 correlates cavitation intensity with vapor pressure for this category of solvents: n‑hexane (20.4 kPa) corresponds to the optimal peak value, with cavitation performance decreasing monotonically on both sides of this maximum.

Next, we examined water-soluble alcohol solvents, including ethylene glycol, 1-propanol, 2-propanol, and ethanol. Following the same procedure, we obtained the dependence of the 476 nm peak intensity on the respective vapor pressure of alcohol solvents. A similar two-way monotonical trend was observed (Fig. 3b and S8). These trends further corroborate that vapor pressure modulates cavitation performance, and that the effects are not limited to Cl-containing solvents.

By comparison, the curve for CCl_4_ displays a relatively sharper peak. This may arise from its high efficiency in generating chlorine radicals, which is related to C-Cl bond dissociation energy. In other words, the combination of high chemical reactivity and near‑optimal vapor pressure is the likely origin of this sharp peak. Surface tension and viscosity are both known to influence cavitation [32], [33]. However, these parameters are not expected to play a dominant role under our conditions, given the low fraction of the organic additives (2.0% v/v). Under the present experimental conditions, vapor pressure clearly serves as the dominant factor, a conclusion that has rarely been highlighted in previous literature.

The observed variation in vapor pressure ranges between Fig. 3a and Fig. 3b may be correlated with solvent solubility. For insoluble n‑hexane, the solution‑phase vapor pressure rises sharply once its concentration exceeds the solubility limit. For miscible isopropanol, by contrast, the vapor pressure of the mixture follows Raoult’s law: at low v/v ratios, the vapor pressure represents a weighted average of the vapor pressures of the individual solvents.

**Fig. 4:**
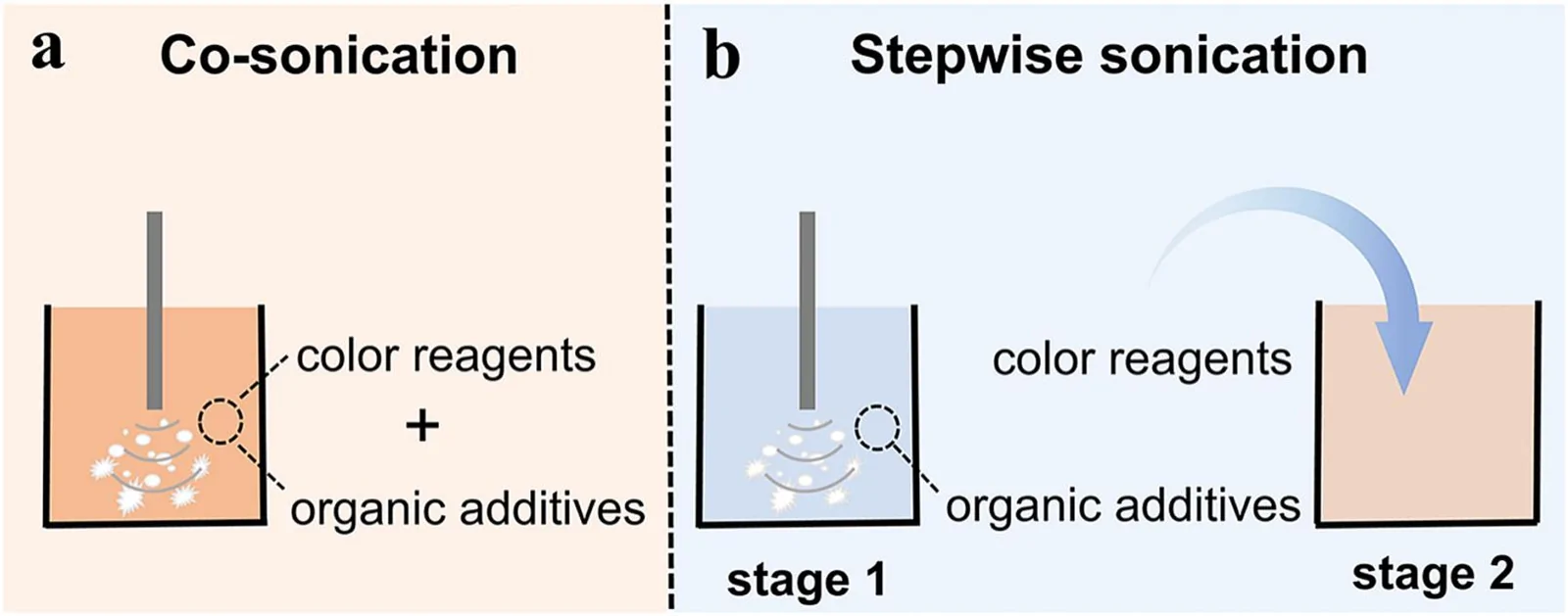
Schematic diagrams of ultrasound experiments: (a) co-sonication experiment; (b) stepwise sonication experiment.

For clarity, the above experiments are termed “co-sonication experiments”, indicating that the color reagents are present during sonication. To investigate the long-lived species after sonication, we designed the following “stepwise sonication experiments” (Fig. 4), so that ultrasonication effects are decoupled from the color reaction: first, an aqueous solution containing a small amount of organic additive was subjected to the standard ultrasonic treatment; subsequently, the color reagents were added to the solution and the color change was quantified.

**Fig. 5:**
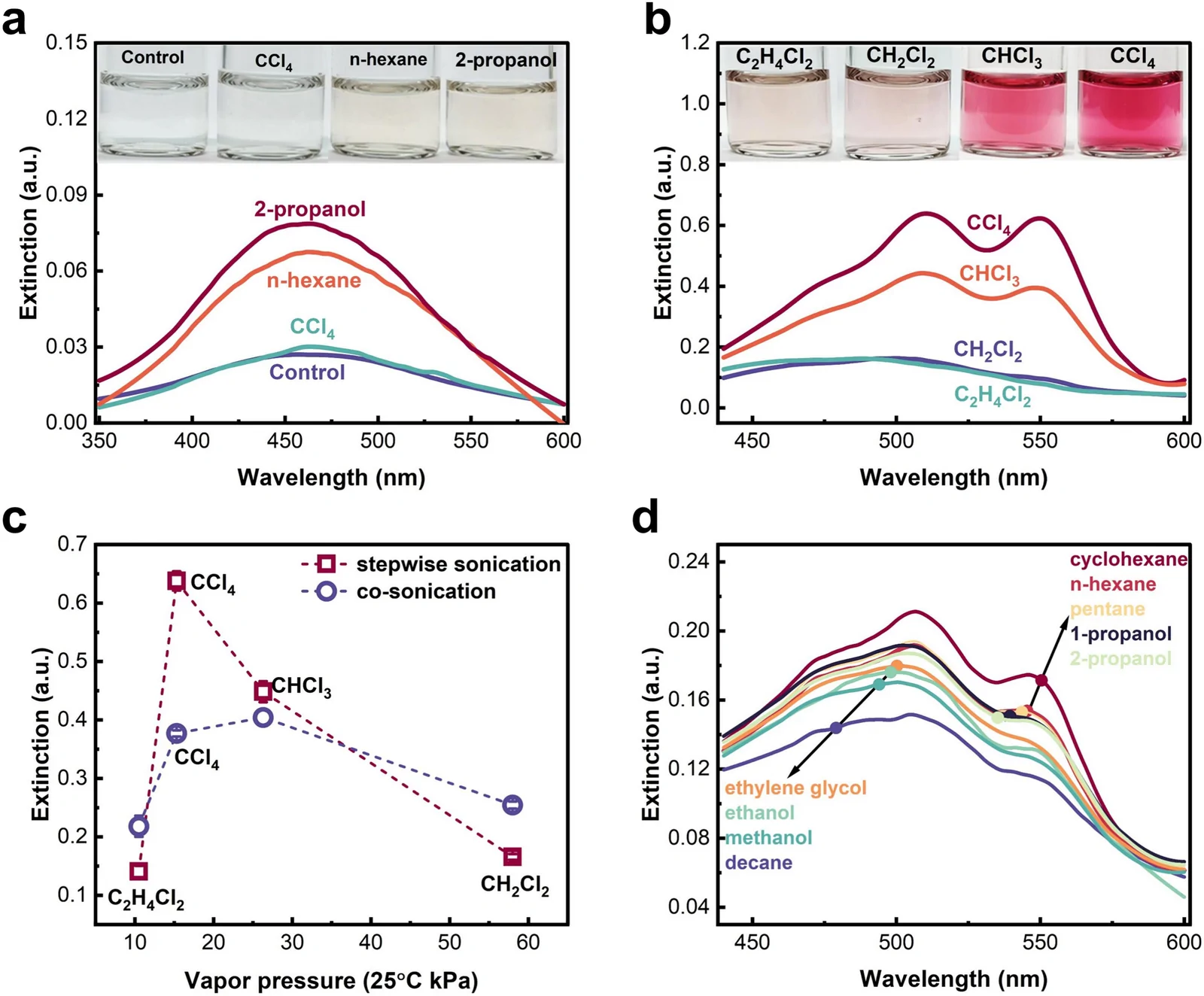
Comparison of stepwise sonication experiments. (a) Comparison of solutions after ultrasonic treatment, followed by addition of [Fe(SCN)_6_]^4−^ reagent (4.50 mM). The control group is pure water without any organic additive, while the other samples contain CCl_4_, n-hexane and 2-propanol as additive, respectively. Inset: Photographs showing the color difference among the four samples after chromogenic reaction; (b) UV–Vis extinction spectra immediately measured after adding DPD reagents to the stepwise sonication experiments promoted by chlorinated solvents. Inset: Color intensity of the C_2_H_4_Cl_2_, CH_2_Cl_2_, CHCl_3_ and CCl_4_-promoted stepwise sonication; (c) comparison of extinction values of the DPD-detected stepwise sonication and co-sonication experiments promoted by chlorinated solvents; (d) color development of DPD‑HRP reagent added to alkane- and alcohol-promoted stepwise sonication experiments.

When CCl_4_ was used as the organic additive, no obvious color variation was detected following post-sonication addition of 4.50 mM (final concentration, same below) [Fe(SCN)_6_]^4−^ (Fig. 5a). However, immediate white flocs occurred upon introducing 0.50 mM AgNO_3_ solution into the freshly sonicated solution (Fig. S1). Control experiments confirmed that CCl_4_ is chemically inert toward AgNO_3_. Therefore, it is reasonable to infer that partial CCl_4_ decomposition had taken place under ultrasonication, generating reactive chlorine-containing byproducts. Specifically, the more reactive chlorine‑containing species (*e.g.*, Cl_2_ and HClO, *vide infra*) formed during sonication gave AgCl precipitate upon reaction with AgNO_3,_ enabling indirect detection of the chlorine‑derived products.

In the literature, N,N-diethyl-*p*-phenylenediamine (DPD) has been reported for the detection of free chlorine, a collective term encompassing Cl_2_, HClO, and their derivatives [34], [35], [36]. Free chlorine oxidizes DPD to form a red compound, which exhibits a characteristic extinction peak at 515 nm. When CCl_4_ was used as the organic additive for sonication (4.0 µL, 0.2% v/v), and the DPD reagent (0.60 mM DPD with 0.50 mM H_2_SO_4_) was added subsequently, a distinct red color was observed (Fig. 5b). This confirms that the sonicated solution did contain oxidizing species. A control experiment of sonicating pure water followed by DPD addition produced no color change. This indicates that oxidizing species such as hydrogen peroxide potentially generated in the aqueous phase are quickly dissipated and thus do not interfere with the detection of free chlorine in this system.

Notably, when the CCl_4_ dosage was increased to 40 µL (2.0% v/v), yellow oily stratification occurred upon mixing the sonicated solution with DPD reagent (Fig. S2), while no such stratification was observed at 4.0 µL (0.2% v/v). Even at the low dosage of 4.0 µL, screening of chlorinated solvents showed that o-chlorotoluene, C_2_H_2_Cl_4_, and chlorobenzene formed yellow oily stratification once mixed with DPD reagent (Fig. S3), likely due to their side reactions with oxidants and derivatives. By contrast, CCl_4_, CHCl_3_, C_2_H_4_Cl_2_, and CH_2_Cl_2_ maintained homogeneous and characteristic red color at a dosage of 4.0 µL.

To evaluate the stability of these unknown oxidizing substances, the CCl_4_ solution after sonication was stored for a given period (*t* = 1 min, 2 h, 4 h, 10 h and 24 h) before the addition of the DPD reagent. The red color was still pronounced and its intensity decreased only slightly (15.3%) over a period of 24 h (Fig. S4a). However, the red color is not stable upon formation, exhibiting 74.7% decrease after merely 10 min storage (Fig. S5). In short, the unknown oxidizing substances are long-lived, but the oxidized form of DPD is not.

Other chlorinated organic solvents including CHCl_3_, C_2_H_4_Cl_2_, and CH_2_Cl_2_ exhibited similar behaviors in stepwise sonication experiments, in terms of giving flocs upon AgNO_3_ addition and red coloration after adding DPD reagent (Fig. 5b and S1). Collectively, these results strongly suggested that the long-lived oxidizing species were free chlorine. For these 4 solvents, the free chlorine production was quantified via the characteristic absorbance at 515 nm, which was plotted against their respective saturated vapor pressure (Fig. 5c). The resulting color intensity against vapor pressure showed a similar volcano-shaped dependence, with relatively higher intensity for CCl_4_ and CHCl_3_, and lower intensity at both ends (Fig. 5c). This trend was consistent with the argument of vapor-pressure-promoted cavitation, but there were obvious differences between co-sonication and stepwise sonication. Specifically, under co‑sonication conditions, the DPD-detected color intensity of the CCl_4_-promoted reaction was approximately 41.3% lower than that obtained under stepwise sonication. This phenomenon can be rationalized by the degradation of DPD chromogenic products induced by reactive chlorine species, although other factors such as direct DPD degradation may also contribute to this behavior.

**Fig. 6:**
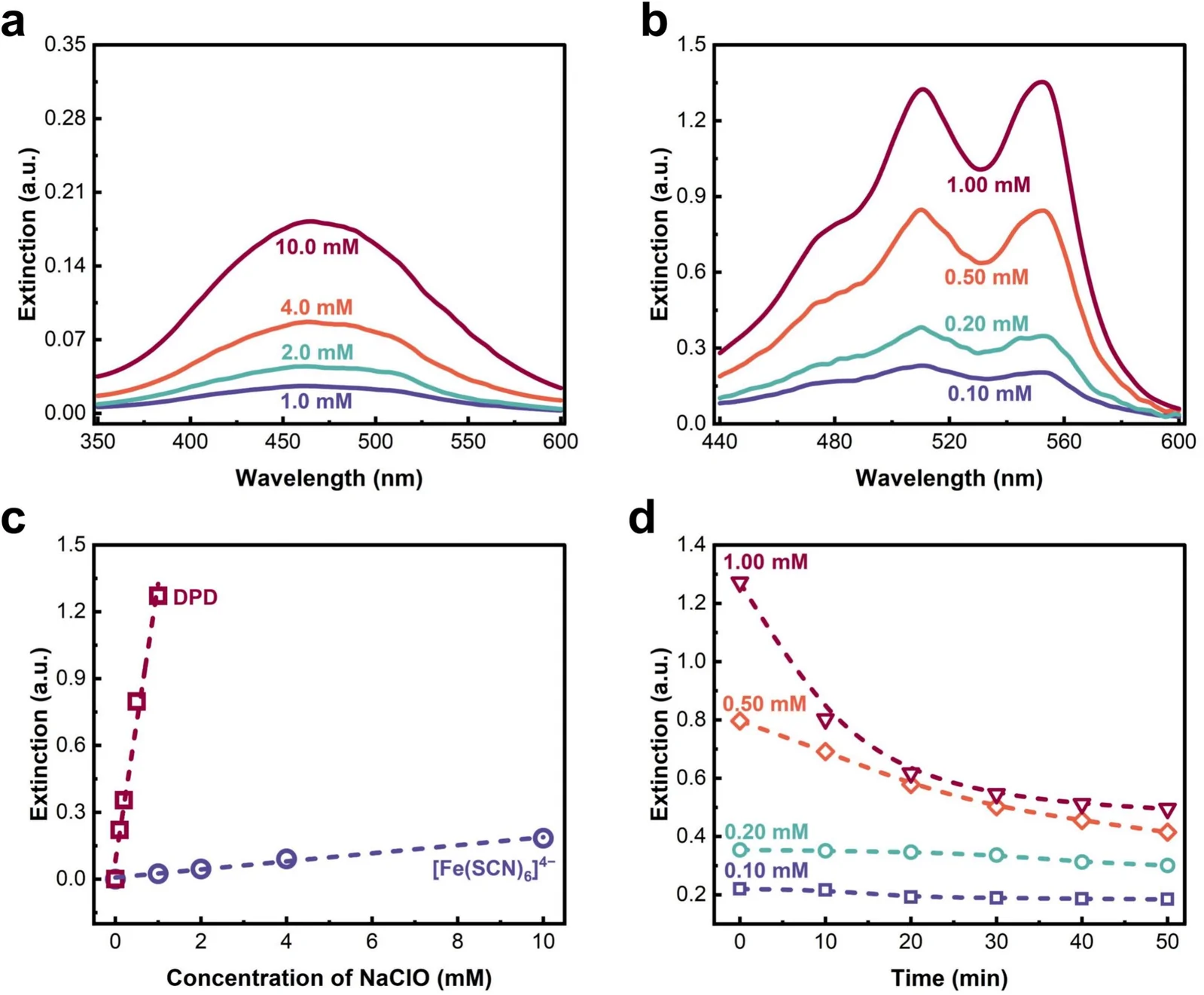
To systematically compare the chromogenic responses between [Fe(SCN)_6_]^4−^and DPD, NaClO was used as the oxidant and the time stability of the oxidized chromogenic product of DPD was investigated. (a) Extinction spectra obtained after mixing [Fe(SCN)_6_]^4−^with 1.0–10.0 mM NaClO solutions; (b) Extinction spectra of DPD reacted with 0.10–1.00 mM NaClO; (c) Variations in characteristic extinction peak intensity of [Fe(SCN)_6_]^4−^ and DPD as a function of NaClO concentration; (d) Time-dependent decay curves of characteristic extinction signals recorded at 515 nm for DPD solutions containing different NaClO concentrations, revealing the instability of the red DPD-derived products.

The instability of DPD in the co-sonication system was verified via control experiments with NaClO. A series of NaClO solutions with concentrations of 0.10, 0.20, 0.50, and 1.00 mM were prepared. For each concentration, 2.0 mL aliquot was mixed with the same amount of DPD reagent. The absorbance variation at 515 nm was immediately recorded using a UV–Visible spectrophotometer (Fig. 6b). Upon mixing, the solution rapidly developed a characteristic red color, which gradually faded. The color fading rate was positively correlated with the NaClO concentration. At a low concentration of 0.1 mM, the red color weakened by 4.6% within 10 min; whereas at 1.0 mM NaClO, the red color weakened by 37% within 10 min (Fig. 6d). As a well-known bleach, NaClO oxidizes and destroys the conjugated system of the DPD. Consequently, DPD is suitable only for immediate detection, as its chromogenic products are prone to degradation and thus, unreliable for long‑term monitoring and specifically, the co‑sonication experiments.

To compare the color reagents [Fe(SCN)_6_]^4−^ and DPD, a series of control experiments were performed using the standard NaClO solutions as the oxidant. As shown in Fig. 6a-c, the color intensity was positively correlated with NaClO concentration in both cases. A concentration of 1.0 mM NaClO only induced negligible color variation with [Fe(SCN)_6_]^4−^, but produced obvious red color with DPD under identical conditions. This confirms that DPD possesses much higher sensitivity for free chlorine detection. On the other hand, [Fe(SCN)_6_]^4−^ reacts with both free chlorine and peroxide-type oxidants (*vide infra*). Despite its lower sensitivity relative to DPD, [Fe(SCN)_6_]^4−^ is better-suited for our co-sonication experiments, as it reflects the overall chemical effects induced by sonication.

After elucidating the active species in chlorinated solvents and the response of two types of color reagents, typical chlorine-free solvents (*e.g.*, n-hexane and 2-propanol) were investigated for comparisons. In stepwise sonication experiments with n-hexane as the additive, immediate addition of [Fe(SCN)_6_]^4−^ after sonication yielded a light red solution (Fig. 5a). When the sonicated solution was stored at room temperature for 24 h before reagent addition, a similar red color was still observed, albeit with a 30.3% decrease in intensity (Fig. S4b). In contrast, parallel tests with DPD reagent showed no characteristic color change. Other chlorine-free solvents, such as 2-propanol, gave similar results as n-hexane (Fig. S6).

These results demonstrated that the oxidizing species generated in chlorine-free solvent systems differ fundamentally from the free chlorine produced in chlorinated systems. Given the long-term stability and oxidative activity of these products, we hypothesized that the dominant species are peroxides. To test this, 0.10 mM H_2_O_2_ and 50.0 mM di-*tert*-butyl peroxide were each reacted with DPD and [Fe(SCN)_6_]^4−^. Neither peroxide triggered red coloration with DPD, but both efficiently reacted with [Fe(SCN)_6_]^4−^ to produce red color. These control experiments matched well with the above observations in chlorine-free sonication systems, supporting the hypothesis of peroxide formation.

It is known in the literature that peroxides cannot directly oxidize DPD, but in the presence of horseradish peroxidase (HRP), they are decomposed into free radicals which in turn oxidize DPD, yielding characteristic absorption at 510 and 551 nm[37], [38]. Accordingly, when we simultaneously added DPD and HRP (0.20 µM) to the n-hexane-promoted stepwise sonication experiment, an immediate red color appeared (Fig. 5d). Replacing n-hexane with 2-propanol or other chlorine-free solvents gave consistent results, confirming that ultrasonic cavitation of chlorine-free solvents produces peroxides.

In the stepwise sonication experiments, the content of active species as quantified by DPD-HRP showed no obvious correlation with vapor pressure (Fig. S9). This discrepancy likely stems from the dependence of DPD assays on HRP, alongside varied peroxide species generated with different additives. Even if ultrasonic cavitation delivers a nearly constant radical generation efficiency (*P*_radical_) across these systems, the activity of radical reaction (*P*_reaction_) differs greatly. These systems produce distinct peroxides, which in turn react with HRP at varying rates. In short, varied intermediates lead to different peroxide products and these factors combined with divergent enzymatic efficiencies, ultimately giving trend-less DPD-HRP detection outcomes.

Similarly, in stepwise sonication experiments, the quantification of active species using the [Fe(SCN)_6_]^4−^ reagent showed no significant correlation with vapor pressure. This is because after standing in stepwise sonication experiments, most short-lived intermediates are rapidly quenched, leaving only peroxides in the solution. With dual redox properties, peroxides tend to undergo spontaneous degradation, and their loss rates vary across different systems. As shown in Fig. 2, the vapor pressure curve measured using [Fe(SCN)_6_]^4−^ in the co-sonication system follows consistent trends, because ultrasonic cavitation generates peroxides as well as short-lived reactive intermediates including hydroxyl radicals, all of which can be captured by [Fe(SCN)_6_]^4−^. Thus, the [Fe(SCN)_6_]^4−^ reagent can deliver stable coloration signals and more meaningful trends. Although the overall capture efficiency of [Fe(SCN)_6_]^4−^ toward reactive species is limited, so long as its capture ratio (*P*_reaction_) remains consistent among all additives, the systems can give meaningful colorimetric trends in the experiments.

Taken together, the experimental results reveal that the active species generated via ultrasonic cavitation differ fundamentally between chlorine-free and chlorinated solvent additives, leading to distinct colorimetric responses from the two types of color reagents (Table 1). Peroxides are the dominant products in chlorine-free solvent systems, and chlorinated solvents mainly yield free chlorine, likely due to their respective chemical reactivity. Such differences in the active components directly account for the divergent color behaviors in the presence of the color reagents. For the DPD assay, peroxides from chlorine-free systems cannot oxidize DPD directly, and the detection relies on enzymatic catalysis by HRP. In comparison, free chlorine formed in chlorinated systems is capable of directly oxidizing DPD to develop color immediately. [Fe(SCN)_6_]^4−^ with a low activation barrier can capture both peroxides and various reactive intermediates.

**Table 1:** Chromogenic tests of reactive species formed with chlorinated/chlorine-free additives under two ultrasonication modes.

Ultrasonic mode	Type of additive	Generated reactive species	[Fe(SCN)_6_]^4−^	DPD (Without HRP)	DPD (With HRP)
Co-sonication	Chlorinated	Radicals and derivatives	✓✓		
	Chlorine‑free		✓✓		
Stepwise sonication	Chlorinated	Free chlorine	✓	✓✓	**−**
	Chlorine‑free	Peroxides	✓	✗	✓

Note: ✓✓Color development, suitable for characterization; ✓Color development, yet not recommended for characterization due to limited detection accuracy of [Fe(SCN)_6_]^4−^ and low catalytic efficiency of HRP; ✗No characteristic color change.

While precise differentiation of the radical generation efficiency (*P*_radical_) and reaction efficiency (*P*_reaction_) remains a great challenge, the consistent trends afforded by [Fe(SCN)_6_]^4−^ highlight its unique merit in balanced detection sensitivity and reliability. Most importantly, the colorimetric trends of both color reagents with the three categories of solvent additives correlate well with additive vapor pressure, supporting our hypothesis that a low solution vapor pressure promotes cavitation by reducing the activation barrier of solution distension and bubble formation.

## 4 Conclusion

In summary, this work studied the chemical effects of ultrasonic cavitation with a focus on the promoting roles of organic additives. Two chromogenic reagents, [Fe(SCN)_6_]^4−^ and DPD, are used to establish an analytical protocol for detecting reactive species in co-sonication and step-wise sonication experiments. The latter was designed to decouple the cavitation process from the chromogenic detection reaction, enabling the investigation of long-lived reactive intermediates. Two categories of reactive species were distinguished: free chlorine was identified as the dominant product in chlorinated solvent systems, while peroxides predominated in alkane- and alcohol-promoted systems. On this basis, the sensitivity and stability of the two chromogenic reagents were compared, clarifying that [Fe(SCN)_6_]^4−^ is more suitable for real-time cumulative detection under co-sonication reactions, whereas DPD, owing to its superior sensitivity for stable intermediates, is more adaptable to stepwise sonication scenarios. The two methods have distinct and complementary advantages, collectively providing a methodological reference for the quantification of reactive intermediates in ultrasonic systems with different organic additives.

Most importantly, by comparing the cavitation effects of chlorinated, alkane, and alcohol organic additives, this study established that the promoting effect of organic additives on cavitation is universal, independent of water solubility or the presence of C-Cl bonds. Experimental measurements revealed the critical dependence on the vapor pressure of the respective organic additives. On this basis, we propose that low vapor pressure reduces the nucleation threshold of cavitation bubbles, thereby enhancing the probability of cavitation, whereas excessively high vapor pressure suppresses intensity of cavitation implosion. Hence, solvent vapor pressure, rather than the chemical reactivity of the additive alone, plays a critical role in modulating ultrasonic cavitation. In contrast to the existing studies, the present work investigates the general principles underlying three categories of solvent additives and elucidates the critical role of additive vapor pressure in promoting ultrasonic cavitation. These understandings will have a broad impact on various ultrasonication-based applications.

## CRediT authorship contribution statement

**Chunhua Wei:** Writing – original draft, Methodology, Investigation, Formal analysis, Data curation. **Yuying Yang:** Validation, Supervision. **Guangyu He:** Validation. **Zhouling Wu:** Investigation. **Hongyu Chen:** Writing – review & editing, Supervision, Funding acquisition, Conceptualization. **Xueyang Liu:** Writing – review & editing, Supervision, Project administration, Funding acquisition, Conceptualization.

## Declaration of competing interest

The authors declare that they have no known competing financial interests or personal relationships that could have appeared to influence the work reported in this paper.

## Appendix A Supplementary data

Supplementary Data 1 Supplementary figures and related experimental information.
