# Supplementary material for: Cavitation enhancement by organic additives: a systematic study probing the universal effect of additive vapor-pressure
Source: Ultrason Sonochem. 2026 Sep 8;133:108043. doi: 10.1016/j.ultsonch.2026.108043 (PMC13583776; doi:10.1016/j.ultsonch.2026.108043)
Supplement: Supplementary file 1 [file mmc1.docx]

**Supporting Information**

Cavitation Enhancement by Organic Additives: A Systematic Study Probing the Universal Effect of Additive Vapor-Pressure

Chunhua Wei,^a,b,c^ Yuying Yang^a,b,c^ Guangyu He,^b,c^ Zhouling Wu,^a,b,c^ Hongyu Chen,^b,c^ and Xueyang Liu,^a,*^

a. Institute of Advanced Synthesis (IAS) and School of Chemistry and Molecular Engineering, Jiangsu National Synergetic Innovation Centre for Advanced Materials, Nanjing Tech University, Nanjing, 211816, China.

b. Department of Chemistry, School of Science, Research Center for Industries of the Future, Westlake University, Hangzhou 310030, P. R. China.

c. Institute of Natural Sciences, Westlake Institute for Advanced Study, Hangzhou 310024, China.

**Fig S1.** Representative photographs of precipitation formed upon the addition of AgNO_3_ solution to chlorinated organic solvents after ultrasonic treatment.

**Fig S2.** The phenomena of different organic solvents (40 µL, 2.0% v/v) mixed with DPD reagent are shown in the figure. Obvious yellow organic phase stratification was observed in the chlorinated organic solvent group (C_2_H_4_Cl_2_, CCl_4_, CHCl_3_, CH_2_Cl_2_) and alkane group (decane, cyclohexane, n-hexane, pentane), while homogeneous solutions were formed in the alcohol groups.

**Fig S3.** Phase separation phenomenon after adding DPD reagent to chlorinated organic solvents (4.0 µL, 0.2% v/v).

**Fig S4.** Time-dependent extinction changes of chromogenic solutions at different standing time after stepwise sonication with organic additives. (a) CCl_4_ system detected by DPD reagent; (b) n-hexane and 2-propanol systems detected by [Fe(SCN)_6_]^4−^ reagent. All samples were measured in triplicate, and error bars are presented for all data points in both panels.

**Fig S5.** Extinction intensity of DPD-colored CCl_4_ aqueous solution after ultrasonic treatment at different standing time intervals. The extinction was measured immediately after color development and subsequently at 1, 2, 4, 6, 8, and 10 min intervals, showing a time-dependent decrease.

**Fig S6.** Extinction spectral responses of the [Fe(SCN)₆]⁴⁻ chromogenic system to three categories of organic solvents under stepwise sonication conditions: (a) chlorinated solvents; (b) alkanes; (c) alcohols.

**Fig S7.** Correlation between the number of C-Cl bonds in organic additives and cavitation performance (characterized by the extinction of the red complex [Fe(SCN)_6_]^3−^) under a constant total molar amount of chlorine atoms of 1.65×10^-4^ mol.

**Fig S8.** Correlation between cavitation performance (characterized by the red complex [Fe(SCN)_6_]^3−^) and the properties of organic additives. (a) Extinction intensity as a function of the number of C-Cl bonds in chlorinated additives. (b) Variation of extinction intensity with C-Cl bond dissociation energy (kJ/mol) for chlorinated additives. (c-e) Extinction intensity as a function of the vapor pressure (at 25 °C) of the respective solvent additives: (c) chlorinated insoluble solvents, (d) alkane solvents, and (e) alcohol solvents. Intermediate points represent mixtures of neighboring solvent additives, and the vapor pressure was estimated by weighted average. (c) o-chlorotoluene(**●**), C_2_H_2_Cl_4_(**▲**), chlorobenzene(**★**), C_2_H_4_Cl_2_(**◆**), CCl_4_(**■**), CHCl_3_(**⬟**), CH_2_Cl_2_(**▼**), mix solvents(**■**); (d) decane(**●**), cyclohexane(**▲**), n-hexane(**■**), n-pentane(**▼**), mix solvents(**■**); (e) ethylene glycol(**●**), 1-propanol(**▲**), 2-propanol(**■**), ethanol(**⬟**), methanol(**▼**), mix solvents(**■**). This figure corresponds to Fig. 2 and Fig. 3 in the main text.

**Fig S9.** Extinction values of characteristic peak measured at 515 nm (a) and 510 nm (b-c) as a function of solvent vapor pressure (25 °C). (a) Chlorinated solvents measured by DPD colorimetric assay under stepwise sonication and co-sonication modes. (b-c) Alkanes and alcohols measured by DPD-HRP assay under stepwise sonication mode. Intermediate data points between pure solvents represent measurements for 1:1 (volume ratio) binary solvent mixtures.

**Fig S10.** The extinction intensity of the red complex [Fe(SCN)_6_]^3−^ as a function of the amount of added (a) n-hexane and (b) 2-propanol in a total volume of 2.0 mL (i.e., 0-3.0% v/v). In the ultrasonic experiments, the total volume of the solution was fixed at 2.0 mL. The labeled value of 0.014 µL/mL corresponds to the solubility limit of n-hexane in water, meaning that a maximum of 0.028 µL of n-hexane can be dissolved in 2.0 mL of water under the experimental conditions. All samples were measured 3 times and error bars are included for all data points, with a few small ones blocked by symbols.

**Colorimetric principle of the [Fe(SCN)_6_]^3−^ reagent[1]**

$$\begin{aligned} 2{Fe}^{2+}+H_{2}O_{2}+2H^{+}=2{Fe}^{3+}+2H_{2}O\#\left( 1 \right) \end{aligned}$$

$$\begin{aligned} {Fe}^{3+}+6{SCN}^{-}=\left[ Fe\left( SCN \right)_{6} \right]^{3-}\#\left( 2 \right) \end{aligned}$$

**Colorimetric principle of the reaction between DPD reagent and H_2_O_2_[2]**

$$\begin{aligned} DPD+H_{2}O_{2}+HRP={DPD}^{\cdot+}\#\left( 3 \right) \end{aligned}$$

**Colorimetric principle of the reaction between DPD reagent and free chlorine[3]**

$$\begin{aligned} DPD+Free chlorine=Red product\#\left( 4 \right) \end{aligned}$$

**Method for calculating bond energy**

Geometry optimizations for the molecule A-B (where A-B = CCl_4_, CH_2_Cl_2_, CH_2_ClCH_2_Cl, CHCl_2_CHCl_2_, CHCl_3_, PheCl or PheClCH_3_) were performed using the Gaussian 16 program package[4] at the B3LYP/def-TZVP level,[5-7] incorporating the IEFPCM water solvation model[8-10] to account for aqueous solvation effects. Additionally, the DFT-D3(BJ) dispersion correction[11, 12] was employed. Subsequent single-point energy calculations for the optimized A-B molecule, as well as the corresponding radicals A· and B·, were carried out at the M06-2X/def2-TZVP level,[13, 14] using the SMD water solvation model[15] and DFT-D3 dispersion correction. Bonding energies were calculated according to the following equation:

$$E_{bonding}=E_{A-B}-E_{A\cdot}-E_{B\cdot}$$

where $E_{A-B}$, $E_{A\cdot}$ and $E_{B\cdot}$ represent the electronic energies of the molecule A-B, the radical A·, and B·, respectively.

**Table S1.** Calculated bonding energies for the selected molecules.

| Species | CCl_4_ | CH_2_Cl_2_ | CH_2_ClCH_2_Cl | CHCl_2_CHCl_2_ | CHCl_3_ | PheCl | PheClCH_3_ |
| --- | --- | --- | --- | --- | --- | --- | --- |
| Bonding Energy(kJ/mol) | 330.8 | 383.8 | 391.5 | 365.2 | 357.3 | 418.5 | 422.4 |

[1] Z. Wu, X. Liu, H. Guo, J. Huang, G. He, H. Chen, X. Liu, Promoting ultrasonic cavitation via Negative-Curvature nanoparticles, Ultrason. Sonochem. 107 (2024) 106924. <https://doi.org/10.1016/j.ultsonch.2024.106924>.

[2] S. Gokulakrishnan, A. Mohammed, H. Prakash, Determination of persulphates using N,N-diethyl-p-phenylenediamine as colorimetric reagent: Oxidative coloration and degradation of the reagent without bactericidal effect in water, Chem. Eng. J. 286 (2016) 223-231. <https://doi.org/10.1016/j.cej.2015.10.058>.

[3] L. Moberg, B. Karlberg, An improved N,N′-diethyl-p-phenylenediamine (DPD) method for the determination of free chlorine based on multiple wavelength detection, Analytica Chimica Acta 407 (2000) 127-133. <https://doi.org/10.1016/s0003-2670(99)00780-1>.

[4] Gaussian 16, Revision C.01, M. J. Frisch, G. W. Trucks, H. B. Schlegel, G. E. Scuseria, M. A. Robb, J. R. Cheeseman, G. Scalmani, V. Barone, G. A. Petersson, H. Nakatsuji, X. Li, M. Caricato, A. V. Marenich, J. Bloino, B. G. Janesko, R. Gomperts, B. Mennucci, H. P. Hratchian, J. V. Ortiz, A. F. Izmaylov, J. L. Sonnenberg, D. Williams-Young, F. Ding, F. Lipparini, F. Egidi, J. Goings, B. Peng, A. Petrone, T. Henderson, D. Ranasinghe, V. G. Zakrzewski, J. Gao, N. Rega, G. Zheng, W. Liang, M. Hada, M. Ehara, K. Toyota, R. Fukuda, J. Hasegawa, M. Ishida, T. Nakajima, Y. Honda, O. Kitao, H. Nakai, T. Vreven, K. Throssell, J. A. Montgomery, Jr., J. E. Peralta, F. Ogliaro, M. J. Bearpark, J. J. Heyd, E. N. Brothers, K. N. Kudin, V. N. Staroverov, T. A. Keith, R. Kobayashi, J. Normand, K. Raghavachari, A. P. Rendell, J. C. Burant, S. S. Iyengar, J. Tomasi, M. Cossi, J. M. Millam, M. Klene, C. Adamo, R. Cammi, J. W. Ochterski, R. L. Martin, K. Morokuma, O. Farkas, J. B. Foresman, and D. J. Fox, Gaussian, Inc., Wallingford CT, (2019).

[5] F.J.D. P. J. Stephens, C. F. Chabalowski and M. J. Frisch, Ab Initio Calculation of Vibrational Absorption and Circular Dichroism Spectra Using Density Functional Force Fields, The Journal of Physical Chemistry 98 (1994) 11623-11627.

[6] A. Schäfer, H. Horn, R. Ahlrichs, Fully Optimized Contracted Gaussian Basis Sets for Atoms Li to Kr, The Journal of Chemical Physics 97 (1992) 2571-2577. <https://doi.org/10.1063/1.463096>.

[7] A. Schäfer, C. Huber, R. Ahlrichs, Fully Optimized Contracted Gaussian Basis Sets of Triple Zeta Valence Quality for Atoms Li to Kr, The Journal of Chemical Physics 100 (1994) 5829-5835. <https://doi.org/10.1063/1.467146>.

[8] E. Cancès, B. Mennucci, J. Tomasi, A New Integral Equation Formalism for the Polarizable Continuum Model: Theoretical Background and Applications to Isotropic and Anisotropic Dielectrics, The Journal of Chemical Physics 107 (1997) 3032-3041. <https://doi.org/10.1063/1.474659>.

[9] B. Mennucci, J. Tomasi, Continuum Solvation Models: A New Approach to the Problem of Solute’s Charge Distribution and Cavity Boundaries, The Journal of Chemical Physics 106 (1997) 5151-5158. <https://doi.org/10.1063/1.473558>.

[10] B. Mennucci, E. Cancès, J. Tomasi, Evaluation of Solvent Effects in Isotropic and Anisotropic Dielectrics and in Ionic Solutions with a Unified Integral Equation Method:  Theoretical Bases, Computational Implementation, and Numerical Applications, The Journal of Physical Chemistry B 101 (1997) 10506-10517. <https://doi.org/10.1021/jp971959k>.

[11] S. Grimme, J. Antony, S. Ehrlich, H. Krieg, A Consistent and Accurate ab initio Parametrization of Density Functional Dispersion Correction (DFT-D) for the 94 Elements H–Pu, The Journal of Chemical Physics 132 (2010) 154104. <https://doi.org/10.1063/1.3382344>.

[12] S. Grimme, S. Ehrlich, L. Goerigk, Effect of the Damping Function in Dispersion Corrected Density Functional Theory, Journal of Computational Chemistry 32 (2011) 1456-1465. <https://doi.org/10.1002/jcc.21759>.

[13] Y. Zhao, D.G. Truhlar, The M06 Suite of Density Functionals for Main Group Thermochemistry, Thermochemical Kinetics, Noncovalent Interactions, Excited States, and Transition Elements: Two New Functionals and Systematic Testing of Four M06-Class Functionals and 12 Other Functionals, Theoretical Chemistry Accounts 120 (2007) 215-241. <https://doi.org/10.1007/s00214-007-0310-x>.

[14] F. Weigend, R. Ahlrichs, Balanced Basis Sets of Split Valence, Triple Zeta Valence and Quadruple Zeta Valence Quality for H to Rn: Design and Assessment of Accuracy, Physical Chemistry Chemical Physics 7 (2005) 3297-3305. <https://doi.org/10.1039/B508541A>.

[15] C.J.C. Aleksandr V. Marenich, and Donald G. Truhlar, Universal Solvation Model Based on Solute Electron Density and on a Continuum Model of the Solvent Deﬁned by the Bulk Dielectric Constant and Atomic Surface Tensions, The Journal of Physical Chemistry B 113 (2009) 6378-6396.
